# Development and pilot testing of a tool to assess evidence-based practice skills among French general practitioners

**DOI:** 10.1186/s12909-018-1368-y

**Published:** 2018-11-09

**Authors:** Nicolas Rousselot, Thomas Tombrey, Drissa Zongo, Evelyne Mouillet, Jean-Philippe Joseph, Bernard Gay, Louis Rachid Salmi

**Affiliations:** 1 Department of General Practice, University of Bordeaux, F-33000 Bordeaux, France; 2 ISPED/Bordeaux School of Public Health, University of Bordeaux, F-33000 Bordeaux, France; 3 Centre INSERM U-1219 Bordeaux Population Health, F-33000 Bordeaux, France; 4 CHU de Bordeaux, Pole de sante publique, Service d’information médicale, F-33000 Bordeaux, France; 5 Département de Médecine Générale, Université de Bordeaux, Case 148. 146 rue Léo Saignat, 33076 Bordeaux cedex, France

**Keywords:** Evidence-based practice, Critical appraisal, Medical education, Kappa reliability, General practice, Skills

## Abstract

**Background:**

There is currently an absence of valid and relevant instruments to evaluate how Evidence-based Practice (EBP) training improves, beyond knowledge, physicians’ skills. Our aim was to develop and test a tool to assess physicians’ EBP skills.

**Methods:**

The tool we developed includes four parts to assess the necessary skills for applying EBP steps: clinical question formulation; literature search; critical appraisal of literature; synthesis and decision making. We evaluated content and face validity, then tested applicability of the tool and whether external observers could reliably use it to assess acquired skills. We estimated Kappa coefficients to measure concordance between raters.

**Results:**

Twelve general practice (GP) residents and eleven GP teachers from the University of Bordeaux, France, were asked to: formulate four clinical questions (diagnostic, prognosis, treatment, and aetiology) from a proposed clinical vignette, find articles or guidelines to answer four relevant provided questions, analyse an original article answering one of these questions, synthesize knowledge from provided synopses, and decide about the four clinical questions. Concordance between two external raters was excellent for their assessment of participants’ appraisal of the significance of article results (K = 0.83), and good for assessment of the formulation of a diagnostic question (K = 0.76), PubMed/Medline (K = 0.71) or guideline (K = 0.67) search, and of appraisal of methodological validity of articles (K = 0.68).

**Conclusions:**

Our tool allows an in-depth analysis of EBP skills, thus could supplement existing instruments focused on knowledge or specific EBP step. The actual usefulness of such tools to improve care and population health remains to be evaluated.

**Electronic supplementary material:**

The online version of this article (10.1186/s12909-018-1368-y) contains supplementary material, which is available to authorized users.

## Background

Evidence-based Practice (EBP) is the integration of best research evidence, clinical expertise, and patient values, in a specific care context [1]. This way of practicing medicine developed in the 1980’s and has subsequently been integrated worldwide within new teaching approaches, centred on problem-based learning. EBP teaching was introduced in many initial and continuing medical education curricula to improve health care by better integrating relevant information from the scientific literature [2–14].

EBP has been described as having five steps [15, 16]: 1) Formulate a clear clinical question about a patient’s problem; 2) Search the literature, with an appropriate strategy, for relevant articles [17]; 3) Critically appraise the evidence for its validity, clinical relevance and applicability; 4) Implement the useful findings back into clinical practice [18]; and 5) Evaluate the impact. This approach is particularly useful in general practice (GP) to manage primary care situations, where it has been described as the sound simultaneous use of a critical research-based approach and a person-centred approach [19, 20].

Whilst many potential advantages have been suggested [16, 21], some criticisms have also been made [22]. A serious drawback is that it has not been clearly shown that EBP can improve physician skills or patient health [23–25]. Very few randomized clinical trials have documented the effect of EBP, with these trials frequently including non-comparable groups. Further, these trials were often based on subjective judgements, due to the lack of reliable and valid tools to assess EBP skills [13, 14, 25–28].

Indeed, some tools have been proposed, but are not easily accessible or validated [14, 28–32]. Most existing tools focus on assessing knowledge, rather than skills, particularly for the literature search [21, 33]; they do not assess skills for each step of EBP [34], but rather focus on article critical assessment [30, 31, 33, 35, 36], sometimes without any relation to a clinical situation [35].

Our aim was to develop a tool to assess the skills necessary for the first four steps of the EBP process, and to evaluate whether independent raters could reliably use the tool to assess acquired skills.

## Methods

**Fig. 1:**
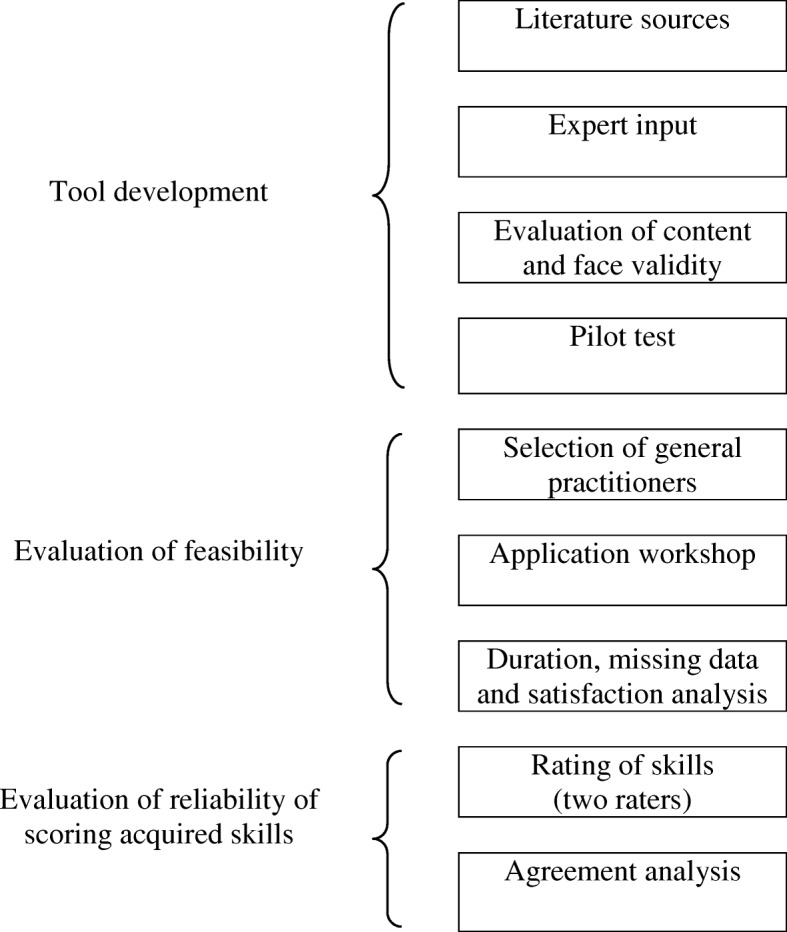
Main steps of EBP skills assessment tool development and testing

To assess EBP skills, we developed a comprehensive tool, including a test of skills and a scoring grid, based on literature and expert advice. We tested the applicability of the test and evaluated whether independent observers could reliably use the scoring tool to analyse answers to the test to assess acquired skills (Fig. 1). Our validity approach was based on a classical model of clinical evaluation of tool validity [37], which provides a strategy to develop and evaluate the performance of tests. This conceptualisation is similar to the “validity as a test characteristic” described in the health professions education literature [38]. This approach is shared in a large part of the French GP teachers who are also clinicians.

### Tool development

#### Literature sources

Our tool was developed based on syntheses of the medical literature on EBP, published in the Journal of the American Medical Association [2, 13, 17, 18, 30, 39–42], and in the British Medical Journal [3, 23, 33, 43]. We also considered previous published tools’ strengths and limitations [29, 33, 34, 36].

#### Expert input on content and purpose of tool

Three of the authors supervised tool development: a senior general practitioner (BG), a senior epidemiologist (LRS), both with recognised experience in EBP teaching in both initial and continuing medical education, and an experienced senior librarian (EM) with experience in teaching literature search for health professionals.

Whereas previous tools mostly assessed knowledge [44], our aim was to assess skills, defined as the participant using knowledge by actually carrying out EBP steps about a clinical scenario [14, 28]. To assess participants’ skills, we asked them to perform tasks associated with the different EBP steps [14], with open but precise instructions, rather than only asking them how they would undertake those tasks. Then, we observed their ability to actually complete these tasks.

We assessed all first four steps of EBP independently, thus allowing participants to undertake all tasks, even if they were wrong in one of the earlier steps. This also allowed participants to receive feedback regarding their results as part of a formative assessment for each step. Our test was also built as a continuum from problems described in a clinical situation to decisions made to deal with these problems. Physician daily constraints (computer and Internet access, time… [45–47]) were also considered when designing the test.

Our tool was divided into four parts to assess necessary skills for each of the first four steps of EBP (Table 1): A clinical vignette (Table 2), on a common and complex situation likely to be seen in primary care, was used to assess the ability to formulate a clear clinical question about a patient’s problem. We asked participants to formulate four clinical questions on diagnostic, prognosis, aetiology, and treatment. The scoring grid for that part was inspired by the first question of the Fresno test [33] and assessed whether the formulated question respected the PICO (Population, Intervention, Comparison, Outcomes) criteria [48]. To assess the ability to search the literature for relevant documents related to the previous clinical questions, we asked participants to find the full text of an original article or guideline for each question. Scoring of this ability was based on recording the participants’ computers screenshots, using the Wink Screen Recording Software 2.0 (available at http://www.debugmode.com/wink/), which registered one screenshot every three seconds during the test. The scoring grid was adapted from a published tool [34] to assess literature search strategies. To assess critical appraisal skills, we selected four English-language full-text original articles, covering each one of the four search questions (diagnostic, prognosis, aetiology, and treatment). Each participant was to appraise the validity of methods, relevance for care, and significance of results of only one of these articles. The scoring grid was based on previous works [1] and specific criteria to appraise the quality of articles on diagnostic [39], prognosis [40], treatment or prevention [41], and harm [42]. To assess the ability to synthesize and decide about a specific clinical situation, we developed four synopses reporting the critical appraisal of the four articles responding to each of the initial clinical questions. The scoring grid assessed clarity of the decision, and elements used to justify the decision, including consideration of the clinical context and a question on the degree to which the participant trusted study results (Additional file 1).

**Table 1:** Main characteristics of the EBP skills assessment tool used for each participant during the test

Test part	EBP step	Task	Support used	Skills: performance assessment^a^
First	Formulate a clinical question	Build 4 search questions to answer a clinical problem	1 case vignette	How complete and relevant are the GPs’ PICO questions?
Second	Search relevant clinical articles	Find 4 relevant articles in medical literature (with different strategies)	4 bibliographic retrieval questions	How thoroughly and efficiently do GP conduct searches?
Third	Critically appraise literature	Appraise validity, relevance and results significance of an article	1 original article	Can GP complete critical appraisals?
Fourth	Implement useful findings in clinical practice	Answer 4 clinical questions	4 synopses (of 4 original articles)	Can GP come to a reasonable interpretation of how to apply the evidence?

*GP* General practitioners ; ^a^according to Tilson et al. [14]

**Table 2:** Summary of the case vignette

A 75-years-old man visits his general practitioner. In his medical history: an ischemic stroke 2 years before, atrial fibrillation, smoking, hypertension, and hypercholesterolemia. He was worried by a risk of epilepsy because of his stroke; asked if his use of coffee was excessive; asked to refill his prescription (with no anticoagulant but aspirin); and complained about a calf pain (without any deep vein thrombosis sign).	

#### Content and face validity

To improve our tool adequacy for its purpose, as part of the “content and face validity” step [37], we asked a panel of experts from the CNGE (French National College of Teachers in General Practice) for a critical review. We asked them to judge the relevance of included items, whether any item was missing, and the format of the tool. Their comments were considered in a pre-test version of the assessment tool and the scoring grid.

#### Pilot test

We tested the assessment tool with a senior GP teacher of the Department of General Practice of Bordeaux and a volunteer second year GP resident, to evaluate its technical applicability and their understanding of instructions. The scoring grids were adapted and filled in once, jointly by two GP raters (TT, DZ), to formalize and homogenize the scoring procedure.

### Evaluation of feasibility

We documented [28, 37]: acceptability of the tool as reflected by participation, number of undocumented items, and satisfaction of participants, time required to complete the test, time required to rate the test; for undocumented items, we tried to judge whether this was related to comprehension or technical problems, for instance failure of the Internet connection.

#### Selection of participants

Participants to a full test were GP residents in internship with general practitioners near Bordeaux, and GP teachers from the Department of General Medicine of Bordeaux. All had a general practice activity and were contacted by phone. Verbal informed consent was obtained from all participants.

#### Application workshop

The test was conducted in computer rooms of the University of Bordeaux, during a three-hour session. Each participant was provided with a computer and Internet access. Once the participants had carried out one part of the process, they sent their output by E-mail to the organizer (TT) and then received instructions for the next part. The first part was expected to last 20 min, i.e. 5 min to formulate each of the four clinical questions. The second part was one-hour long, i.e. 15 min to search one document. Each participant had to find four documents: two original articles using PubMed/MEDLINE, one document using research tools to specifically identify guidelines, and one document using a free search on the Web. The order in which participants were to find the different types of documents was randomly allocated, so that three faculty and three residents were searching in the same order. The third part was 45-min long. Each participant had to analyse one of four articles. Here again the article was randomly allocated so that each type of article was analysed by three faculty and three residents. The last part was 40-min long, i.e. 10 min to analyse each of the four synopses and write the decision.

#### Duration, missing data and satisfaction analysis

The duration of tests and scoring was measured and missing or ambiguous data analysed. An anonymous satisfaction questionnaire (Additional file 2) was filled in by participants at the end of the test. After the test, participants received a synopsis of what was expected from them.

### Evaluation of reliability of scoring acquired skills

#### Rating of acquired skills

Two of the authors (TT, DZ) independently corrected all anonymized tests, filling the scoring grids. They judged, on a four-level Likert scale the conformity of output to what was expected to reflect a given skill (for example, completely conform to expected PICO; rather conform; rather not conform; completely not conform). They separately scored: each of the four clinical questions; each of the three search strategies; appraisal of the methodological validity, relevance for care, and significance of results; each of the four decisions (Table 3, Additional file 1).

**Table 3:** Results of Likert scales for each assessed task of the EBP steps

Step	NC	Completely conform		Rather conform		Rather not conform		Completely not conform	
	n	n	%	n	%	n	%	n	%
Formulating a focused question									
Diagnostic	1	0	0.0	3	13.0	9	39.1	10	43.5
Prognosis	1	2	8.7	7	30.4	5	21.7	8	34.8
Etiologic	1	1	4.3	15	65.2	3	13.0	3	13.0
Therapeutic	1	0	0.0	2	8.7	15	65.2	5	21.7
Best information search									
PubMed/MEDLINE	8	0	0.0	0	0.0	4	17.4	11	47.8
Guidelines	17	0	0.0	3	13.0	3	13.0	0	0.0
Free search (Web)	4	4	17.4	4	17.4	7	30.4	3	13.0
Critical appraisal									
Methodological validity	3	1	4.3	2	8.7	9	39.1	8	34.8
Relevance for patient care	3	0	0.0	1	4.3	12	52.2	7	30.4
Significance of results	3	0	0.0	0	0.0	4	17.4	16	69.6
Synthesis and decision									
Diagnostic article	1	1	4.3	8	34.8	12	51.2	1	4.3
Prognostic article	1	13	56.5	4	17.4	5	21.7	0	0.0
Etiologic article	1	12	51.2	5	21.7	5	21.7	0	0.0
Therapeutic article	2	5	21.7	10	43.5	3	13.0	3	13.0

*n* = number of participants, *NC*= not completed (missing data)

#### Agreement analysis

Analyses were done from data where neither the participant nor the rater was identified, with the SAS statistical software package, version 9.0 (SAS Institute Inc.). A linear weighted Kappa coefficient and its 95% confidence interval (CI) was calculated for each Likert scale to measure concordance between the two assessments [37]. Kappa was considered excellent if higher than 0.8, good if between 0.6 and 0.8, medium if between 0.4 and 0.6, and low if under 0.4 [49]. The main analysis considered missing data as completely not conform. A second analysis excluded missing data. An analysis of the sources of discrepancies between the two raters was done collegially, with the two raters and a senior epidemiologist (LRS).

## Results

### Feasibility

#### Selection of participants

Of the 28 general practice residents who were contacted, 12 agreed to participate. Of the 85 GP teachers of the Department of General Practice of Bordeaux, 46 could be contacted by phone, and 14 agreed to participate; three withdrew after initially agreeing, including one who cancelled three days before the workshop and could not be replaced. Eventually, 12 GP second-year residents, two men and 10 women, and 11 GP teachers, 10 men and one woman, participated. The GP teachers were one associate professor, three assistant professors and seven part-time instructors; they were aged 53 years on average.

#### Test and scoring duration

The workshop followed all steps as planned. The average response time was 171 min for teachers and 185 min for residents. There was a difference in the last part of the workshop (33 min for teachers and 44 min for residents), and the set time was exceeded for the third part of the test (53 min for teachers and 56 min for residents). The scoring lasted on average 44 min by test for the first rater (total: 17 h), and 30 min by test for the second rater (total: 11 h 50 min).

#### Missing data

Data on the test was missing in 14.6% of the Likert scales, 16.9% for teachers and 12.5% for residents (Table 3). Most missing data was for the second part of the test: four of the 23 participants’ computer screenshot files were lost (3 for teachers), possibly due to handling errors by participants. Such errors were also seen once in the first part, three times in the third part, and once in the last part. Instructions were not followed for bibliographic retrieval for 17 of the 69 Likert scales scored: 11 for residents; four were for PubMed/MEDLINE and 13 for guideline searches.

#### Satisfaction

Satisfaction questionnaires were filled by 22 participants. All participants were satisfied: they found the experience interesting (100%), relevant (82%), useful for clinical practice (100%), but difficult (97%). They expressed that the workshop underscored the need for training (91%) and the tool assessed well participant familiarity with EBP (91%) and could be used to assess progress with training (86%). Only 46% reported using EBP in their usual practice with the main reasons for not using it being: lack of time (94%), poor understanding of English (59%) and lack of skills to use necessary tools (71%).

### Reliability of acquired skills scoring

#### Agreement analysis

Concordance between the two raters was excellent for their assessment of participants’ appraisal of the significance of article results (Table 4). It was good for the formulation of a diagnostic question, PubMed/Medline or guideline search, and for methodological validity appraisal. It was lower for all other aspects.

**Table 4:** Concordance between the two raters’ Likert scale for each question of the EBP steps

Step	Agreement		Weighted Kappa (K)		Weighted Kappa excluding missing data	
	n	%	K	95% CI	K	95% CI
Formulating a focused question						
Diagnostic	19	82.6	0.76	0.53–0.99	0.75	0.51–0.99
Prognosis	13	56.5	0.58	0.36–0.81	0.56	0.33–0.79
Etiologic	13	56.5	0.40	0.07–0.72	0.34	−0.00-0.68
Therapeutic	13	56.5	0.32	−0.02-0.65	0.27	−0.08-0.61
Best information search						
PubMed/MEDLINE	21	86.7	0.75	0.42–1.00	0.71	0.34–1.00
Guidelines	22	83.3	0.93	0.79–1.00	0.67	0.10–1.00
Free search (Web)	13	47.4	0.58	0.31–0.85	0.39	0.10–0.67
Critical appraisal						
Methodological validity	18	78.3	0.68	0.40–0.95	0.72	0.47–0.97
Relevance for patient care	17	73.9	0.59	0.32–0.86	0.53	0.23–0.83
Significance of results	22	95.7	0.83	0.51–1.00	0.83	0.50–1.00
Synthesis and decision						
Diagnostic article	11	47.8	0.21	−0.01-0.44	0.23	−0.04-0.50
Prognostic article	9	39.1	0.45	0.24–0.65	0.39	0.20–0.59
Etiologic article	9	39.1	0.27	0.00–0.53	0.26	0.07–0.45
Therapeutic article	12	52.2	0.44	0.19–0.70	0.37	0.11–0.63

*n* = number of participants with agreement between raters, *CI* = confidence interval

The main sources of discrepancy were: differences in appreciation of PICO criteria (the difference between an “incomplete” and “not conform” response depending on response precision, which was not assessed equally by the two raters); raters’ entry errors and irrelevant response not scored as “not conform”; errors and omissions in filling scoring grid; discrepancies in assessment of articles and website quality for free research; differences in appreciation of decision making and synthesis, depending on rater’s harshness and expectation for decisions to be explained. In case of disagreement between raters, we chose to keep the most favourable assessment for this last question only.

## Discussion

We developed the first French-language tool to assess EBP skills of general practitioners. Concordance between raters was excellent for assessment of the participants’ appraisal of the significance of article results. It was good for the formulation of a diagnostic question, PubMed/MEDLINE and guideline searches, and for article methodological validity appraisal. It was lower for all other aspects.

Our tool covers all relevant skills, as the main four steps of the EBP process are assessed. In that regard, it completes existing tools, such as the Fresno test [33] and the Berlin questionnaire [36], as both only include the first three steps, and focus mostly on critical appraisal [14]. The only published validated test assessing those four steps is the ACE tool [21]. Our tool is again complementary, as the ACE tool assesses more knowledge than skills, using simple true-false questions, whereas our tool includes observation of actual searches and critical appraisals. This more focused assessment of knowledge rather than skills is also a limitation of the Fresno test, which mostly covers literature search and critical appraisal, and of the Berlin Questionnaire.

We assessed physicians’ skills with open-ended questions, asking for the completion of specific tasks; for instance, our observation was innovative with the recording of screenshots, and assessed them with objective items. These features make our tool and its application closer to and more relevant for clinical practice. It has been developed using various kinds of complex questions relating to real-life situations, which, to our knowledge, has not been done before; we believe it could be transposed to many complex clinical situations.

We still have to improve parts of the tool before in can be proposed to the EBP teaching community. Concordance between raters was low, notably for the last part of the test related to synthesis and decision making. More precise scoring grids and a better application of assessment items are needed to reduce raters’ subjectivity when assessing skills. This was also sometimes seen for the first part of the test, regarding formulation of a search question. This first part, based on the Fresno test for which good inter-rater reliability has been documented [33], was composed of questions on short and simple case vignette. This part of the Fresno test had a low variability of possible responses, whereas our test was closer to practice.

Another potential limitation of our test is the time needed for its completion; three hours, much longer than the ACE tool and Berlin Questionnaire (15–20 min), and Fresno test (one-hour long) [21, 33, 36]. Simplifying our tool might shorten this completion time, but is likely to reduce its relevance for practice. Moreover, time devoted to each part (5 min to build a search question, 15 min to find an original article, 45–60 min to analyse it, and 10 min to synthesize and decide) is a realistic reflection of what can be done in practice.

Two possible reasons for the low level of reliability of some items of our tool are the low level of skills, and the variation in the harshness of raters. Another hypothesis is that the tool is not a valid reflection of the actual skills. Indeed, a tool well-perceived by users (the so-called “face validity”), of which the content has been agreed by experts (content validity) and which showed acceptable reliability, might still not adequately measure what it is supposed to measure [37, 50]. Therefore, we still need studies of the construct or criterion validity of our tool. However, the latter is difficult to assess, as there is no gold standard for all EBP skills. A gold standard could be developed through expert judgement based on formal consensus methods [51].

As our tool yields 14 independent scores, it is well suited to identify which of the skills a student or a physician should focus his future training on (formative assessment). However, we still need to develop a way to provide profiles for the four main skills and a judgment of an individual’s overall EBP skills, as a way to compare participants and evaluate our tool’s validity. Other perspectives to further develop our test and evaluate its performance should take into consideration limitations of our study: small number of testers, precluding the use of other analytical techniques to evaluate reliability such as log linear models.

As our work was initiated by the GP Department of the University, we selected participants with a practical experience in GP. Indeed, we wanted to assess the ability to use EBP skills to improve patients care in a GP setting. Moreover, the use of the same clinical scenario throughout the whole assessment process is an indirect way to evaluate the potential impact of acquired skills in clinical practice. We also selected GP residents and teachers to get a heterogeneous sample, as recommended to evaluate reliability [52]. Nevertheless, we believe, by looking at the responses, that all residents were probably not EBP fledglings and all GP teachers, given their age, were not EBP experts, as already shown elsewhere [45]. This generation contrast, the small number of participants and raters [53], and the focus on a population linked with the University probably limit the generalizability of our results.

## Conclusions

Our tool is relevant for practice as it allows an in-depth analysis of EBP skills. It could respond to a real need to better assess EBP skills of general practitioners. It can also be seen as usefully complementing existing tools, but further validation, including comparison with the latter, is needed. The actual usefulness of such tools to improve care and population health remains to be evaluated.

## Additional files

Additional file 1: Two parts of the tool to assess EBP skills: 1) Content of the skill assessment form; and 2) Scoring grid. This file gives more information about our tool. (DOCX 61 kb)

Additional file 2: Satisfaction questionnaire. This file presents the satisfaction questionnaire filled in by participants at the end of the test. (DOCX 16 kb)

## Abbreviations

EBP: Evidence-based practice
GP: General practice
