# Supplementary material for: Development and pilot testing of a tool to assess evidence-based practice skills among French general practitioners
Source: BMC Med Educ. 2018 Nov 9;18:254. doi: 10.1186/s12909-018-1368-y (PMC6234795; doi:10.1186/s12909-018-1368-y)
Supplement: Supplementary file 1 — Two parts of the tool to assess EBP skills: 1) Content of the skill assessment form; and 2) Scoring grid. This file gives more information about our tool. (DOCX 61 kb) [file 12909_2018_1368_MOESM1_ESM.docx]

**Additional file 1:** **Two parts of the tool to assess EBP skills: 1) Content of the skill a****ssessment form; and 2) Scoring grid.**

**1) Skill assessment form**

***First part: Case vignette***

Participants had 20 minutes to read a case vignette and answer four questions.

*Summary of the case vignette:*

A 75-years-old man visits his general practitioner. In his medical history: an ischemic stroke 2 years before, atrial fibrillation, smoking, hypertension, and hypercholesterolemia. He was worried by a risk of epilepsy because of his stroke; asked if his use of coffee was excessive; asked to refill his prescription (with no anticoagulant but aspirin); and complained about a calf pain (without any deep vein thrombosis sign).

*Questions:*

All participants had to write four clinical questions about issues raised during this visit, in order to search the literature and find relevant information for the care of this patient.

- Question 1 had to be on a diagnostic issue.
- Question 2 had to be on a prognosis issue.
- Question 3 had to be on an aetiology issue.
- Question 4 had to be on a treatment issue.

Participants had to feel in a document and send it by mail to the organizer. Then, they received the second part of the test.

***Second part: Literature search***

Provided with a free Internet access, participants had to find the full text of an original article. They had 15 minutes by search. Participants’ computers screenshots were registered during the test.

- For a diagnostic problem, the proposed bibliographical search question was: for a primary care patient with a suspicion of deep vein thrombosis, is Wells score, with or without D-dimers, valid compared with the gold standard of Doppler ultrasonography, to exclude a deep vein thrombosis?
- For a prognosis problem, the proposed bibliographical search question was: for a 75-years-old man with a history of ischemic stroke, is the epilepsy risk increased?
- For an aetiology problem, the proposed bibliographical search question was: for a 75-years-old man who smoke, and has hypertension and hypercholesterolemia, does coffee increase the risk of epilepsy?
- For a treatment problem, the proposed bibliographical search question was: for elderly people with atrial fibrillation who had a stroke, is warfarin more effective than aspirin to avoid a new stroke?

For each of these bibliographical questions, participants had to use the following search strategy. They all had to find the full text of an original article (or a literature review), using a PubMed/Medline search twice, with a free search once, and to find a guideline once. The order in which participants had to use the different strategies was randomly allocated, so that three faculty and three residents were searching in the same order (four groups).

Participants had to send the four articles full text by mail to the organiser. Then, they received the third part of the test.

***Third part: Critical appraisal***

Participants had 45 minutes to read one provided original article and to carry the following tasks:

- to judge the validity of methods.
- to judge the relevance for the care of the patient described in the vignette (see above).
- to judge the study results significance.

No more information was given to participants.

One of the four proposed articles was randomly allocated to each participant so that each type of article (diagnostic [1], prognosis [2], etiologic [3] or therapeutic [4] issue -was analysed by three faculty and three residents.

Participants had to feel in a document and send it by mail to the organizer. Then, they received the last part of the test.

***Fourth part: Synthesis and decision***

All participants had 40 minutes to read four given synopses of the four original articles [1-4], and state what they would decide for the vignette’s patient for each of the for clinical issues.

The four synopses synthesized articles and described the study population, procedures, main measures, main results, conflicts of interest, and main biases.

The participants had to state and justify their decisions. Then, they had to feel in a document and send it by mail to the organizer.

***Analysed articles:***

1. Oudega R, Hoes AW, Moons KGM. The Wells rule does not adequately rule out deep venous thrombosis in primary care patients. Ann Intern Med. 2005;143(2):100–7.

2. Burn J, Dennis M, Bamford J, Sandercock P, Wade D, Warlow C. Epileptic seizures after a first stroke: the Oxfordshire Community Stroke Project. BMJ. 1997;315(7122):1582–7.

3. Larsson SC, Männistö S, Virtanen MJ, Kontto J, Albanes D, Virtamo J. Coffee and tea consumption and risk of stroke subtypes in male smokers. Stroke. 2008;39(6):1681–7.

4. Mant J, Hobbs FDR, Fletcher K, Roalfe A, Fitzmaurice D, Lip GYH, et al. Warfarin versus aspirin for stroke prevention in an elderly community population with atrial fibrillation (the Birmingham Atrial Fibrillation Treatment of the Aged Study, BAFTA): a randomised controlled trial. Lancet. 2007;370(9586):493–503.

**2) Scoring grid**

Rater:

Participant number:

Time starting scoring:

Time at end of scoring:

***Question 1: clinical question formulation***

For each of the four questions:

|  | Conform | Incomplete | Not conform | Absent | NA |
| --- | --- | --- | --- | --- | --- |
| Population description is |  |  |  |  |  |
| Intervention description is |  |  |  |  |  |
| Comparison description is |  |  |  |  |  |
| Outcomes description is |  |  |  |  |  |

*Conform: described by one or several precise words.*

*Incomplete: described by a single precise word if several words are expected.*

*Not conform: described by a not precise word.*

*Absent: if the search term is not described.*

*Not Applicable (NA): comparison for prognosis and aetiology.*

*Different PICOs were possible (list in a help to raters).*

***Question 2: Bibliographic search.***

For each of the two PubMed/Medline Search:

|  | Yes | No |
| --- | --- | --- |
| At least three search keywords provided |  |  |

|  | Correct | Not conform | No |
| --- | --- | --- | --- |
| Search method is documented |  |  |  |

*Correct: when use of the Clinical Queries filter (for all question type)*

*Not conform: when another way was used.*

|  | Yes | No |
| --- | --- | --- |
| Filters used |  |  |

|  | Yes | No |
| --- | --- | --- |
| MeSH Terms used |  |  |

If Yes:

|  | Yes | | No |
| --- | --- | --- | --- |
| Subheadings used |  | |  |
| MeSH Major Topic used |  | |  |
| Number of MeSH Terms used | |  | |

|  | Yes | No | Relevant chosen abstract | No article | DNK |
| --- | --- | --- | --- | --- | --- |
| Relevant chosen article |  |  |  |  |  |

*Relevant (when reading the abstract): population similar to patients seen by French general practitioners, important results, main outcome relevant for clinical practice, well described and available for physicians to be able to innovate in their practice.*

|  | < 5 min | 5 < t < 10 min | 10 < t < 15 min | > 15 min |
| --- | --- | --- | --- | --- |
| Time spent |  |  |  |  |

For free search:

|  | Level 1 | Level 2 | Level 0 | DNK |
| --- | --- | --- | --- | --- |
| Relevance level of the first visited website |  |  |  |  |

*Level 1: Synopsis: Minerva, Critique et Pratique, EBM journal, DARE, ACP journal Club, BMJ EBM, Bandolier… Systematic reviews: Cochrane library, Ovid ebmr... EBM Filters: PubMed Clinical Queries, Tripdatabase, Sumsearch, Google scholar EBM. Prescrire. Systems: Clinical Evidence, Uptodate, Clinical Knowledge Summaries, PIER.*

*Level 2: Search engine or directory:* *Science direct, Doccismef, Drefc. English-speaking journals of good quality (original articles): JAMA, NEJM, The Lancet, BMJ, CMAJ…*

*Level 0: Google, Umvf, EMC, Esculape. Francophone journals (mainly didactic articles): Revue médicale suisse, Revue médicale belge, La revue du praticien, Le médecin du Québec, La revue médicale de Bruxelles, Le généraliste…*

| Number of irrelevant websites visited |  |
| --- | --- |

|  | Yes | No | No article | DNK |
| --- | --- | --- | --- | --- |
| Relevant chosen article |  |  |  |  |

|  | Yes | No |
| --- | --- | --- |
| English language |  |  |

|  | < 5 min | 5 < t < 10 min | 10 < t < 15 min | > 15 min |
| --- | --- | --- | --- | --- |
| Time spent |  |  |  |  |

For guideline search:

|  | National or similar institution | Learned society or consensus conference | Patient association | DNK |
| --- | --- | --- | --- | --- |
| Guideline authors/origin |  |  |  |  |

*National or similar institution: for example: HAS, NICE, NGCH…*

|  | Yes | No | DNK |
| --- | --- | --- | --- |
| Relevance: guideline answers the question |  |  |  |

|  | A | B | C | DNK |
| --- | --- | --- | --- | --- |
| Evidence level |  |  |  |  |

|  | Yes | No |
| --- | --- | --- |
| Have methods been seen? |  |  |

|  | Yes | No |
| --- | --- | --- |
| English language |  |  |

|  | < 5 min | 5 < t < 10 min | 10 < t < 15 min | > 15 min |
| --- | --- | --- | --- | --- |
| Time spent |  |  |  |  |

***Question 3: Critical appraisal.***

*Diagnostic article:*

Methods validity.

Has the participant consistently mentioned these characteristics in order to assess the methodological validity of the study?

|  | Yes | Not completely | No | DNK |
| --- | --- | --- | --- | --- |
| Study design is consistent with the aim of the study |  |  |  |  |
| Patients’ selection is described and appropriate, disease frequency is compatible with epidemiological data |  |  |  |  |
| Withdrawals are indicated, explained and reasonable |  |  |  |  |
| The patient sample includes an appropriate spectrum of patients to whom the diagnostic test will be applied in clinical practice |  |  |  |  |
| Independent and blind comparison with a reference standard |  |  |  |  |
| The reference standard was performed whatever were the results of the test being evaluated. |  |  |  |  |
| The reference standard was measured with the same (valid and reliable) methods for all subjects |  |  |  |  |
| The test is reliable (reproducible for the same observer and by different observers). |  |  |  |  |
| The test was validated in a second independent patient group. |  |  |  |  |
| Ethical rules have been respected |  |  |  |  |
| Other features?  Specify: _______________________________ |  |  |  |  |

*Yes: if the idea formulated is clearly and precisely stated.*

*Not completely: if a part of the expected idea is missing or if the idea presented is not clear.*

*No: if the idea formulated does not appear in the analysis.*

Relevance for care.

Has the participant consistently mentioned these characteristics in order to assess the relevance of the study?

|  | Yes | Not completely | No | DNK |
| --- | --- | --- | --- | --- |
| The test is available in his/her medical practice |  |  |  |  |
| The test is economically accessible in his/her medical practice |  |  |  |  |
| The test is accurate and precise in his/her conditions of medical practice |  |  |  |  |
| Consequences of the test can help his/her patient care (there is a treatment) |  |  |  |  |
| It is a frequent or serious disease |  |  |  |  |
| Performing conditions of the test are sufficiently described for it to be applicable |  |  |  |  |
| His patient is relatively similar to the patients included in the study |  |  |  |  |
| Other features?  Specify: __________________________________ |  |  |  |  |

Significance of results.

Has the participant consistently mentioned these characteristics in order to assess the significance of the study results?

|  | Yes | Not completely | No | DNK |
| --- | --- | --- | --- | --- |
| Accuracy parameters are mentioned: LR, Se, Sp, PPV, NPV |  |  |  |  |
| Accuracy parameters are reported with their confidence intervals |  |  |  |  |
| Test results are / are not important enough to make a clinical decision |  |  |  |  |
| Sample size was calculated before study and is sufficient |  |  |  |  |
| Other features?  Specify: |  |  |  |  |

*Prognostic article:*

Methods validity.

Has the participant consistently mentioned these characteristics in order to assess the methodological validity of the study?

|  | Yes | Not completely | No | DNK |
| --- | --- | --- | --- | --- |
| Study design is coherent with the aim of the study |  |  |  |  |
| Representative and well-defined sample of patients |  |  |  |  |
| Patients are at a similar point in the course of the disease. The starting point is well defined |  |  |  |  |
| Patients follow up sufficiently long and complete |  |  |  |  |
| Blind or objective measure of outcome event |  |  |  |  |
| Outcome is measured in the same way, with a reliable and valid method |  |  |  |  |
| Comparability between groups is maintained: withdrawals are indicated, explained, reasonable and balanced |  |  |  |  |
| Results were validated in an independent sample of patients compared to the study sample |  |  |  |  |
| Ethical rules have been respected |  |  |  |  |
| Other features?  Specify: __________________________________ |  |  |  |  |

Relevance for care.

Has the participant consistently mentioned these characteristics in order to assess the relevance of the study?

|  | Yes | Not completely | No | DNK |
| --- | --- | --- | --- | --- |
| His/her patient is relatively similar to the patients included in the study |  |  |  |  |
| The primary endpoint is clinically relevant |  |  |  |  |
| It is a frequent or serious disease |  |  |  |  |
| Prognostic factors are easily measurable, identifiable and clear (clinical practical feasibility) |  |  |  |  |
| Clinical impact of data on what is going to be proposed or told to the patient (the patient can benefit from it) |  |  |  |  |
| Other features?  Specify: |  |  |  |  |

Significance of results.

Has the participant consistently mentioned these characteristics in order to assess the significance of the study results?

|  | Yes | Not completely | No | DNK |
| --- | --- | --- | --- | --- |
| Statistical significance is defined by p <0.05 or by absence of reference value in the CI |  |  |  |  |
| Probability of outcome events over time is mentioned by a RR |  |  |  |  |
| Prognosis accuracy estimates are given with the confidence interval |  |  |  |  |
| Test results are / are not important enough to make a clinical decision |  |  |  |  |
| Sample size was calculated before study and is sufficient |  |  |  |  |
| Statistical methods used are appropriate |  |  |  |  |
| An adjustment for the known confounders has been made |  |  |  |  |
| Statistical significance was judged on the primary endpoint. The Scheffé or other method was used if multiple comparisons |  |  |  |  |
| Other features?  Specify: __________________________________ |  |  |  |  |

*Treatment article:*

Methods validity.

Has the participant consistently mentioned these characteristics in order to assess the methodological validity of the study?

|  | Yes | Not completely | No | DNK |
| --- | --- | --- | --- | --- |
| Study design is consistent with the aim of the study |  |  |  |  |
| Described and appropriate subjects’ selection. Eligible subjects are representative of the target population. |  |  |  |  |
| Computer-based randomization |  |  |  |  |
| Centralized randomization (blinded assignment) |  |  |  |  |
| Patients, health workers and study personnel blind to treatment |  |  |  |  |
| Similar groups at the start of the trial |  |  |  |  |
| Comparability is maintained in groups: withdrawals are indicated, explained, reasonable and balanced |  |  |  |  |
| Groups treated equally aside from the experimental intervention |  |  |  |  |
| Patients follow-up sufficiently long and complete (no withdrawals) |  |  |  |  |
| Reliable and valid measurement of the main outcome |  |  |  |  |
| All patients analysed in the groups to which they were randomized |  |  |  |  |
| Ethical rules have been respected |  |  |  |  |
| Other features?  Specify: __________________________________ |  |  |  |  |

Relevance for care.

Has the participant consistently mentioned these characteristics in order to assess the relevance of the study?

|  | Yes | Not completely | No | DNK |
| --- | --- | --- | --- | --- |
| His/her patient is relatively similar to the patients included in the study |  |  |  |  |
| Possible use of the treatment in the patient's environment (availability, cost, monitoring...) |  |  |  |  |
| Treatment use is sufficiently detailed |  |  |  |  |
| It is a frequent or serious disease |  |  |  |  |
| Main outcome clinically important and relevant |  |  |  |  |
| Favourable benefit / risk balance of the treatment |  |  |  |  |
| Other features?  Specify: ___________________________________ |  |  |  |  |

Significance of results.

Has the participant consistently mentioned these characteristics in order to assess the significance of the study results?

|  | Yes | Not completely | No | DNK |
| --- | --- | --- | --- | --- |
| Statistical significance is defined by p <0.05 and by absence of reference value in the CI |  |  |  |  |
| The effect size is documented by a RRR, ARR or NNT |  |  |  |  |
| The precision of estimates is given by a confidence interval |  |  |  |  |
| Test results are / are not important enough to make a clinical decision |  |  |  |  |
| Sample size was calculated before study and is sufficient |  |  |  |  |
| Statistical methods used are appropriate |  |  |  |  |
| An adjustment for the known confounders has been made |  |  |  |  |
| Statistical significance was judged on the primary endpoint. The Bonferroni or other method was used if multiple comparisons |  |  |  |  |
| Other features?  Specify: ____________________________________ |  |  |  |  |

*Harm article:*

Methods validity.

Has the participant consistently mentioned these characteristics in order to assess the methodological validity of the study?

|  | Yes | Not completely | No | DNK |
| --- | --- | --- | --- | --- |
| Study design is consistent with the aim of the study |  |  |  |  |
| Described and appropriate subjects’ selection. |  |  |  |  |
| Clearly identified comparison groups that were similar with respect to important determinants of outcome, other than the one of interest |  |  |  |  |
| Blind and objective measure of outcomes or exposures, in the same way in the groups being compared |  |  |  |  |
| Patient follow up sufficiently long (for outcome to occur) and complete (no withdrawals) |  |  |  |  |
| Comparability is maintained in groups: withdrawals are indicated, explained, reasonable and balanced |  |  |  |  |
| Results on the adverse effects respect causality criteria (dose-response gradient, prior exposure, biological consistency, other study, discontinuation) |  |  |  |  |
| Ethical rules have been respected |  |  |  |  |
| Other features?  Specify: ___________________________________ |  |  |  |  |

Relevance for care.

Has the participant consistently mentioned these characteristics in order to assess the relevance of the study?

|  | Yes | Not completely | No | DNK |
| --- | --- | --- | --- | --- |
| Unfavourable benefit-risk ratio of the causal agent |  |  |  |  |
| His/her patient is relatively similar to the patients included in the study |  |  |  |  |
| Exposure to the causal agent is quite common in his/her patients |  |  |  |  |
| Main outcome clinically important and relevant |  |  |  |  |
| Other features?  Specify: ____________________________________ |  |  |  |  |

Significance of results.

Has the participant consistently mentioned these characteristics in order to assess the significance of the study results?

|  | Yes | Not completely | No | DNK |
| --- | --- | --- | --- | --- |
| Statistical significance is defined by p <0.05 and by absence of 1 in the CI |  |  |  |  |
| The strength of the association between exposure and outcome is given by the RR or OR |  |  |  |  |
| The precision of estimations of the association between exposure and outcome is given by the confidence interval |  |  |  |  |
| Test results are / are not important enough to make a clinical decision |  |  |  |  |
| Statistical methods used are appropriate |  |  |  |  |
| Sample size was calculated before study and is sufficient |  |  |  |  |
| An adjustment for the known confounders has been made |  |  |  |  |
| Statistical significance was judged on the primary exposure. Otherwise, the multiple comparisons problem has been considered |  |  |  |  |
| Other features?  Specify: |  |  |  |  |

***Question 4: Synthesis and decision making.***

For each of the four decisions:

Has the participant decided to apply the conclusions of the article?

Are the facts presented in the synopsis considered relevant and valid for the patient care, i.e. is there trust in the results?

|  | Yes with confidence | Yes with restrictions | No | Do not know / No answer |
| --- | --- | --- | --- | --- |
| Diagnostic article |  |  |  |  |
| Prognostic article |  |  |  |  |
| Treatment article |  |  |  |  |
| Harm article |  |  |  |  |

*It is assessed if the participant has restrictions about the presented facts or if he has a total confidence in it; or if he thinks results are not valid or relevant to be applied to his patient.*

**Help for rating: Determination of level for Likert scales.**

***Question 1: clinical question formulation***

Completely conform: 4 correct or incomplete (or 3 true or incomplete for prognosis and aetiology).

Rather conform: 3 correct or incomplete.

Rather not conform: 2 correct or incomplete.

Completely not conform: 1 or 0 correct or incomplete.

***Question 2: Bibliographic search.***

*For each PubMed/Medline search:*

Completely conform: 4 tools used.

Rather conform: 3 tools used.

Rather not conform: 2 tools used.

Completely not conform: 1 or 0 tool used.

Tools = At least three search keywords present; search method documented; filters used; MeSH terms used.

*For free search:*

Completely conform: relevant article and level 1 or 2 website visited.

Rather conform: relevant article and level 0 website visited.

Rather not conform: no article found or not relevant and level 1 or 2 website visited.

Completely not conform: no article found or not relevant and level 0 website visited.

*For guideline search:*

Completely conform: 3 criteria met.

Rather conform: 2 criteria met.

Rather not conform: 1 criterion met.

Completely not conform: no criterion met.

Criteria = official guideline, methods have been seen, answer the question.

***Question 3: Article critical appraisal.***

*Methods validity:*

Completely conform: 4 criteria met.

Rather conform: 3 criteria met.

Rather not conform: 2 criteria met.

Completely not conform: 1 or 0 criterion met.

Criteria = study design, participant selection, ethics

+ main outcome (for treatments and prognosis) or the test (for diagnostic) or causality criteria (for harm).

*Relevance for care:*

Completely conform: 3 criteria met.

Rather conform: 2 criteria met.

Rather not conform: 1 criterion met

Completely not conform: 0 criterion met

Criteria = similar patients, available treatment, favourable benefit / risk balance (for treatment); similar patients, practice feasibility, benefit for patient (for prognosis); similar patients, relevant main outcome, benefit / risk balance (for harm); similar patients, available test, reliable test, usefulness (diagnostic: cf rating with four criteria as methods validity).

*Significance of results:*

Completely conform: 4 criteria met.

Rather conform: 3 criteria met.

Rather not conform: 2 criteria met.

Completely not conform: 1 or 0 criterion met.

Criteria = statistical significance (CI, p), clinical significance (RR or OR), sample size, relevant statistical methods (for treatment, prognosis and harm). For diagnostic: 3 criteria (cf relevance rating) = test parameters, CI, clinical significance.

***Question 4: Synthesis and decision making.***

Completely conform: if correct facts interpretation and conform decision.

Rather conform: if correct facts interpretation but not conform decision.

Rather not conform: if facts interpretation is not correct but conform decision.

Completely not conform: if facts interpretation is not correct and not conform decision.

*There was a help for rating for this question (with examples of correct interpretations and decision making for the case vignette).*
