# Supplementary material for: Development and pilot testing of a tool to assess evidence-based practice skills among French general practitioners
Source: BMC Med Educ. 2018 Nov 9;18:254. doi: 10.1186/s12909-018-1368-y (PMC6234795; doi:10.1186/s12909-018-1368-y)
Supplement: Supplementary file 2 — Satisfaction questionnaire. This file presents the satisfaction questionnaire filled in by participants at the end of the test. (DOCX 16 kb) [file 12909_2018_1368_MOESM2_ESM.docx]

**Additional file 2: Satisfaction questionnaire.**

1. Are you satisfied with having participated in this study?

□ Very satisfied □ Rather satisfied □ Rather not satisfied □ Not at all satisfied

2. What have you thought of this study?

- Relevant for practice □ Yes □ Rather □ Not very □ No

- Interesting (you learned something) □ Yes □ Rather □ Not very □ No

- Difficult □ Yes □ Rather □ Not very □ No

- Helpful to identify training needs □ Yes □ Rather □ Not very □ No

- Other: ………………………………………………………………...................................

3. Do you think we have tried to measure your familiarity with EBP, that is, the tool is a good reflexion of your EBP skills and limitations?

□ Absolutely □ Rather yes □ Rather no □ Not at all

4. Do you think this tool could be used to evaluate progress in a training process (in the context of continuing medical education for example)?

□ Absolutely □ Rather yes □ Rather no □ Not at all

5. Is the EBP decision-making approach (4 steps), as analysed in this study, relevant to your daily practice?

□ Absolutely □ Rather yes □ Rather no □ Not at all

If not, what are the reasons?

…………………………………………………………………………………………………...

6. Are you used to the EBP approach in daily clinical practice?

□ Absolutely □ Rather yes □ Rather no □ Not at all

If not, what are the barriers to apply this approach in daily clinical practice?

□ Lack of time

□ Bad understanding of medical English

□ Lack of knowledge of necessary tools

□ Lack of skills to use necessary tools

□ Lack of interest

□ Other: ……………………………………………………………………………………..
